# Building-related illness (BRI) in all family members caused by mold infestation after dampness damage of the building

**DOI:** 10.3205/dgkh000403

**Published:** 2021-12-07

**Authors:** Axel Kramer, Thomas A. Wichelhaus, Volkhard Kempf, Michael Hogardt, Kai Zacharowski

**Affiliations:** 1 Institute of Hygiene and Environmental Medicine, University Medicine Greifswald, Germany; 2 Institute of Medical Microbiology and Infection Control, University Hospital Frankfurt, Frankfurt/Main, Germany; 3 University Center of Competence for Infection Control of the State of Hesse, Frankfurt/Main, Germany; 4 Department of Anesthesiology, Intensive Care Medicine and Pain Therapy at the University Hospital Frankfurt, Goethe University, Frankfurt/Main, Germany

**Keywords:** dampness damage, molds infestation, building related illness, symptomatology, course of the disease, influencing factors

## Abstract

**Introduction:** In 2010, dampness damage in a single-family house caused a massive mold infestation. In the further course, the 5 family members developed severe health problems. This report investigates the extent and cause of the water damage. In addition, the various visible fungal infestations were analyzed in a specialized laboratory.

**Results:** Due to building construction errors, starting from the basement, an increased moisture penetration of the residential building was detected.

Within 2 years, massive mold infestation occurred. In 2016, the following species were detected: *Cladosporium sphaerospermum, Chaetomium globosum, Penicillium chrysogenum, Scopularis brevicaulis, Acremonium furculum, A. charticola *and* A. sclerotigenum, Trichomonascus apis Aspergillus versicolor *and* Debaryomyces hansenii*. Additionally, different black molds were macroscopically detected.

The severity of the disease process varied, probably due to the different daily exposure of the family members, and possibly influenced by age. The children presented acute episodes with nocturnal cough, associated with sleep disturbances and respiratory infections with severe rhinitis. In addition, general fatigue was noticeable. The course of the disease was complicated by recurrent nightly nosebleeds. The mother developed a much more severe course as chronic fatigue syndrome. Additionally, the following continuous complaints occurred: sore throat and headache, nocturnal irritable cough, chronic rhinitis, difficulty concentrating, increasing forgetfulness and word-finding disorders, cognitive impairment with reduced short-term memory, extremely dry eyes with red sclerae, morning stiffness, dyspnea, disturbed temperature regulation (chills), increased feeling of thirst, and menstrual disorders. The father’s building-related illness (BRI) was comparatively mild due to much lower exposure, with nocturnal irritable cough, rhinitis, and marked fatigue. In 2018, after moving out of the house, the father was symptom-free after 2 weeks, the three children after 6 months, but the mother only after 18 months.

**Discussion:** The symptoms are consistent with reports from the literature, according to which fatigue, sleep disturbances, lack of concentration and headache as well as recurrent infections of the upper respiratory tract are caused by microbial volatile organic compounds (MVOCs) released by molds. The association with recurrent nosebleeds in childhood has not been described in this form before.

**Conclusion:** Since in all family members complete remission of symptoms occurred after cessation of the 6-year exposure, there is no doubt that the BRI was caused by the massive mold infestation.

## Zusammenfassung

**Einleitung:** Im Jahr 2010 kam es auf Grund eines Feuchteschadens in einem Wohnhaus zu massivem Schimmelpilzbefall und im weiteren Verlauf bei der 5-köpfigen Familie zu zunehmenden gesundheitlichen Beschwerden. Aus diesem Anlass sollte eine Ursachenklärung mit Ermittlung des Ausmaßes des Wasserschadens und Differenzierung des optisch sichtbaren Pilzbefalls durchgeführt werden.

**Ergebnisse:** Es konnte ausgehend vom Kellergeschoss eine baulich bedingte zunehmende Durchfeuchtung des Wohnhauses nachgewiesen werden.

Innerhalb von 2 Jahren kam es zu massivem Schimmelpilzbefall. Im Jahr 2016 wurden folgende Species nachgewiesen: *Cladosporium sphaerospermum, Chaetomium globosum, Penicillium chrysogenum, Scopularis brevicaulis, Acremonium furculum, A. charticola, d A. sclerotigenum, Trichomonascus apis Aspergillus versicolor *und* Debaryomyces hansenii*. Zusätzlich waren makroskopisch verschiedene Schwarzschimmelspecies vertreten.

Die Erkrankung war unterschiedlich schwer ausgeprägt, vermutlich bedingt durch die unterschiedliche tägliche Exposition der Familienmitglieder und möglicherweise altersabhängig beeinflusst. Bei den Kindern traten die Beschwerden in Form akuter Schübe mit nächtlichem Husten, verbunden mit Schlafstörungen und respiratorischen Infektionen mit starker Rhinitis auf. Zusätzlich war eine generelle Ermüdung auffällig. Der Krankheitsverlauf wurde durch nächtlich auftretendes rezidivierendes Nasenbluten kompliziert. Bei der Mutter entwickelte sich eine deutlich schwerere Verlaufsform in Form eines Chronic Fatigue Syndroms. Zusätzlich traten folgende Dauerbeschwerden auf: Hals- und Kopfschmerzen, nächtlicher Reizhusten, chronische Rhinitis, Konzentrationsschwierigkeiten, zunehmende Vergesslichkeit und Wortfindungsstörungen, kognitive Einschränkungen mit reduziertem Kurzzeitgedächtnis, extrem trockene Augen mit roten Skleren, morgendliche Steifigkeit, Dyspnoe, gestörte Temperaturregulierung (Frösteln), vermehrtes Durstgefühl und Störungen der Menstruation. Beim Vater war das BRI wegen deutlich geringerer Exposition vergleichsweise gering ausgeprägt mit nächtlichem Reizhusten, Rhinitis und ausgeprägter Müdigkeit. Nach dem Wechsel der Wohnung waren der Vater nach 2 Wochen, die drei Kinder nach 6 Monaten und die Mutter erst nach 18 Monaten beschwerdefrei.

**Diskussion:** Das Beschwerdebild deckt sich mit Berichten aus der Literatur, wonach durch von Schimmelpilzen freigesetzte MVOCs Müdigkeit, Schlafstörungen, Konzentrationsschwäche und Kopfschmerz sowie rezidivierende Infektionen der oberen Atemwege verursacht werden. Die Vergesellschaftung mit rezidivierendem Nasenbluten im Kindesalter ist in dieser Form bisher nicht beschrieben.

**Schlussfolgerung:** Da es nach der Unterbrechung der insgesamt über 6 Jahre währenden Exposition zur kompletten Remission der Beschwerden kam, gibt es keinen Zweifel daran, dass die BRI durch den massiven Schimmelpilzbefall ausgelöst worden ist.

## Introduction

The usual rapid occupation of a building or dwelling after new construction or renovation, often associated with inadequate drying and ventilation, but above all due to the reduced air exchange through insulating exterior cladding and tightly closed windows, often leads to compromised well-being and even health complaints in the form of sick building syndrome (SBS), when the indoor air is contaminated by increased outgassing of volatile organic compounds (VOCs). 

SBS was first defined by the WHO in 1982 [1]. While in in the past, mainly sulfur dioxide, carbon monoxide and dioxide, nitrogen oxides, airborne dusts and polycyclic aromatic hydrocarbons were released by stove heating, open fireplaces and other combustion processes, today it is mainly the VOCs released from building materials, varnishes, paints, adhesives, wallpapers, floor coverings, carpets, furniture and natural wood which are responsible for the pollution of indoor air. In workplaces, additional influencing factors can be toner dust, work climate, psychosocial stress and/or one-sided VDUxx work and/or electromagnetic stress. SBS can also be caused by the release of microbial volatile organic compounds (MVOCs) from molds after extensive indoor mold infestation or by biofilm formation on filters in air conditioning systems. How humans react to polluted indoor air depends not only on the extent of exposure but also on different factors of the immediate environment, such as noise, lighting, indoor climate, illumination, cigarette smoke and vapors, but also on gender, age, nicotine abuse, allergic diathesis and other dispositional factors [2], [3], [4], [5], [6], [7], [8], [9], [10], [11]. Because it is often a summation effect, determining the cause can be difficult in individual cases. Typical symptoms after long-term exposure to VOCs or MVOCs are mucous membrane irritation of eyes, nose, and throat, headache, unusual tiredness or fatigue, and, less frequently, dry or itchy skin [9], [10], [11]. It is typical that the symptoms subside shortly after leaving the living or working quarters [6].

Building-related illness (BRI) can be manifested by symptomatology similar to that of SBS, but is medically more serious and is caused by exposure to detected noxae in the room or building [12]. However, molds have the peculiarity that no dose-response relationship is known, because sensitivity to them varies greatly individually [13], [14]. Four main types of BRI are distinguished: allergic and immunological diseases (e.g., hypersensitivity pneumonia, sinusitis, building-related asthma), irritation (e.g., dry eyes, irritant-induced asthma), infections (e.g., Pontiac fever and Legionnaires' disease), and damage from chemicals and other substances (e.g., radon, asbestos) [15]. Mold infestation after dampness damage are common exposures in buildings and are frequently associated with building-related symptoms [15].

The following is a description of a severe BRI that affected all family members with varying severity after dampness damage in a single-family dwelling.

## Methods

### Medical evaluation of the health status 

In an initial interview in 2012, the complaints were recorded by a specialist in hygiene and environmental medicine in individual discussions with each of the family members. At the same time, the mold infestation was visually assessed. A renewed investigation took place in 2016 and thereafter annually up to 2020.

### Identification of the water damage

A comprehensive inspection of building dampness was performed by experts for subsoil assessment in 2012.

### Mold diagnostics

In 2017, samples from visibly infested walls were transferred into sterile NaCl-containers. Samples were inoculated on sabouraud glucose agar (Oxoid, Wesel, Germany), malt extract agar (Oxoid), and columbia sheep blood agar (Oxoid). Cultures were incubated for seven days at 30°C and a further seven days at room temperature (approx. 20°C). Grown colonies were subcultivated on sabouraud glucose agar. Fungal DNA from pure cultures was extracted via High Pure PCR Template Preparation Kit (Roche, Mannheim, Germany) and subjected to molecular analysis by means of amplification and sequencing of the internal transcribed spacer (ITS) DNA situated between the small-subunit ribosomal RNA (rRNA) and large-subunit rRNA genes, as described by White et al. [16]. For species identification, gene sequences were subjected to the BLAST online tool in the NCBI Genbank database.

## Results

### Characterization of moisture damage

In the fall of 2010, the family house was completed. Immediately after moving in, there was water ingress into the basement and later in the same year, severe moisture damage with subsequent infestation of initially black-looking mold first in the basement, and then in the winter of 2016/2017 also in the sleeping area on the 1^st^ floor. It turned out that large quantities of stratum water (not groundwater) flowing to the house could not be led away from there, but were held in the basement. This resulted in a permanent build-up of water. Consequently, the subsoil was softened and lost its load-bearing capacity, which led to movements in the building structure. Investigations revealed that old, unprofessional, existing drainage systems had been partly reused and that newly constructed drainage systems had also been partly installed unprofessionally.

In 2012, the following massive construction defects were identified:

- Lack of a soil survey prior to the start of construction
- the existing pipes were not checked before or during the construction phase
- the external basement staircase was not dismantled during construction of the additional floor slab; it acted as a funnel and reservoir for incoming water
- incorrect design of the drainages and thus, over the years, the accumulation/pressure of water on the building
- unsuitable basement exterior insulation for stagnant/pressurized water
- no surface drainage of the floor slab
- use and connection of wastewater pipes without testing them according to DIN 1946-6 and 18017; some pipes were leaky or were mixed up, so that rainwater and wastewater were mixed together
- incorrect dimensions of the ventilating system.

During subsequent remediation work, it was found that insufficient suitable backfill material had been used. Instead, the clay soil excavated on site was backfilled. The area around the house had not been adequately compacted. In consequence, the base insulation was demolished, the sidewalks were lowered, and the house moved.

Although dehumidifiers had been operated regularly since 2010 to reduce the high humidity, there was significant mold growth in most of the rooms in the basement. In 2016, the non-functionaldrainage system was replaced. During the winter months, most of the exterior walls of the basement were left open or not sealed. In the winter of 2016/ 2017, this led to changes in the dew point in the basement wall, resulting distribution of water vapor throughout the house also on the upper floor, which led to additional water vapor pollution, especially in the sleeping area on the upper floor. This resulted in massive mold infestation in the bedroom of the 3 children and the parents, as well as the walk-in closet.

### Mold infestation

For the isolated species *Cladosporium sphaerospermum* [17], *Chaetomium globosum* [18], *Penicillium chrysogenum* [19] and *Scopularis brevicaulis* [20], the pathogenic and allergenic potency has been clearly established. *Aspergillus versicolor*, which was also isolated, is considered a moisture indicator. It produces hepatotoxic and carcinogenic mycotoxins and has high irritant potential for eyes, nose, throat/lung and can cause allergies [20], [21], [22], [23]. The isolated mold species *Acremonium furcatum* and *A. sclerotigenum* may be pathogenic [24]. In contrast, *Debaryomyces hansenii* is widespread in natural habitats, occurs in various cheeses, is a component of the human resident flora, and does not appear to have pathogenic significance [25]. For the detected mold species *Trichomonascus apis*, the human pathogenic significance is unclear. Since infestations with black mold species of variously pronounced phenotypes were visually evident, *Aspergillus* spp. such as *A. brasiliensis* and* A. niger* may also have been present. Likewise, *Alternaria alternata* may also have been involved.

Since undeniable health complaints in the form of BRI associated with long-term residential mold infestation are sufficient for the evaluation of causality, comprehensive species identification was not mandatory, since all black mold species release microbial volatile organic compounds (MVOCs), such as alcohols, aldehydes, aromatics, esters, alkanes, furans, ketones, terpenes, and sulfur compounds into indoor air [26], [27].

### Complaint pattern

At the time of the visually manifest mold infestation in 2012, the two boys were 6 and 4 years old. The 11-month-old girl was exposed to the health hazard in the particularly vulnerable phase after birth.

Exposure resulted in health impairments of varying severity, with the children’s symptomatology manifesting more in acute episodes. Both boys suffered from recurrent nosebleeds that generally occurred once a week, and in subsequent years, up to four times a week, primarily at night during sleep. In 2018, this symptomatology occurred only six times per month in the older boy and at least twice per month in the younger boy. The girl also suffered from recurrent nosebleeds starting in 2012, but only in distributive annual intervals. Starting in 2013, the three children regularly experienced nocturnal cough associated with sleep disturbances and recurrent respiratory infections (6–8 times per year) with severe rhinitis and general fatigue. Concentration difficulties became noticeable in both boys beginning in 2013.

The mother (born 1975) manifested a severe course. As of 2012, the symptomatology was characterized by excessive physical and mental exhaustion and severe chronic fatigue syndrome, regularly occurring sore throat 5–6x/year, severe headaches several times a month, chronic rhinitis, severe concentration difficulties, increasing forgetfulness, word-finding disorders, cognitive impairment, partial amnesia with reduced short-term memory, extremely dry eyes, morning stiffness, dyspnea, disturbed temperature regulation (shivering), increased feeling of thirst (diabetes mellitus was ruled out) and, as of 2015, menstrual disorders with irregular as well as increased intermenstrual bleeding. Especially the chronic rhinitis and sneezing, which is very pronounced in winter, in connection with strongly reddened red sclerae, suggest an allergic reaction. After elimination of the fungal infestation in the mother’s wardrobe in April 2018, the symptoms improved slightly.

Since the father was only at home for a relatively short time every day and frequently on the road, he was significantly less exposed than the rest of the family. Therefore, his BRI was only mild. When present in the house, there was nocturnal irritable cough, rhinitis, and marked fatigue the following day.

Until changing their place of residence in 2018, the complaints were present. Following the move into another home. the father was free of all symptoms after 2 weeks, the three children after 6 months, but the mother only after 18 months.

## Discussion

The complaint pattern is consistent with reports in the literature that MVOCs released by molds cause fatigue, sleep disturbances, poor concentration, and headache, as well as recurrent upper respiratory tract infections [9], [10], [11]. Due to the high, long-term exposure, the course in the children was aggravated by recurrent nosebleeds, which has not been previously reported in the literature. There is only a reference to the possibility of triggering nosebleeds on the web page of a construction office for building biology technology [28]. 

## Conclusions

Since a complete remission occurred in all family members after cessation of the mold exposure lasting about 6 years, there is no doubt that the BRI was caused by the mold exposure.

## Notes

### Competing interests

The authors declare that they have no competing interests.
